# Comparison of two surgical interventions for lumbar brucella spondylitis in adults: a retrospective analysis

**DOI:** 10.1038/s41598-023-43812-5

**Published:** 2023-10-04

**Authors:** Dingyu Jiang, Liang Ma, Xiyang Wang, Zhenchao Xu, Guannan Sun, Runze Jia, Yunqi Wu, Yilu Zhang

**Affiliations:** 1 Department of Spine Surgery and Orthopaedics, Xiangya Hospital of Central South University, 87# Xiangya Road, Changsha, 410008 Hunan China; 2 Hunan Engineering Laboratory of Advanced Artificial Osteo-Materials, 87# Xiangya Road, Changsha, 410008 Hunan China; 3 Department of Orthopedics, The Eighth Affiliated Hospital of Xinjiang Medical University, No. 106 Yan’an Road, Tianshan District, Urumqi City, Xinjiang Uygur Autonomous Region, Urumqi, 830049 China

**Keywords:** Neuroscience, Diseases, Neurology

## Abstract

This retrospective study aimed to compare the clinical efficacy of the posterior procedure with the combined anterior and posterior procedure in the surgical management of lumbar Brucella spondylitis. From January 2015 to June 2020, a total of 62 patients presenting with lumbar Brucella spondylitis underwent either one-stage posterior pedicle fixation, debridement, and interbody fusion (Group A, n = 33) or anterior debridement, bone grafting, and posterior instrumentation (Group B, n = 29). All patients were followed up for an average of 25.4 ± 1.5 months and achieved complete resolution of lumbar Brucella spondylitis. No significant differences between the groups were observed in terms of age or pre-operative, three-month postoperative and final follow-up indices of the VAS, ESR, CRP, lordosis angle, ODI scores, fusion time, and time of serum agglutination test conversion to negative (*P* > 0.05). Each patient exhibited notable improvements in neurological function, as assessed by the JOA score rating system. Group A demonstrated significantly shorter operative duration, intraoperative blood loss, and hospital stay compared to Group B (*P* < 0.05). Superficial wound infection was observed in one case in Group A, whereas Group B experienced one case each of intraoperative peritoneal rupture, postoperative ileus, iliac vein injury, and superficial wound infection. This study supports the efficacy of both surgical interventions in the treatment of lumbar Brucella spondylitis, with satisfactory outcomes. However, the posterior approach demonstrated advantages, including reduced surgical time, diminished blood loss, shorter hospital stays, and fewer perioperative complications. Consequently, the one-stage posterior pedicle fixation, debridement, and interbody fusion represent a superior treatment option.

## Introduction

Brucellosis, a zoonosis caused by *Brucella*, has resulted in an increasing health burden and economic loss for humans and livestock industries in China^1,2^. Furthermore, the incidence of human brucellosis has been rapidly increasing in recent years^3,4^. As a systemic illness, brucellosis can affect multiple organ systems, with the musculoskeletal system being among the most frequently involved. The lumbar spine, in particular, represents the most susceptible site for Brucella infection^5–7^. Intervertebral disc destruction is the primary etiology of Brucella spondylitis whilevertebral destruction and paravertebral abscesses are relatively infrequent^6^. The diagnosis of lumbar Brucella spondylitis (LBS) relies on characteristic manifestation, an epidemiological exposure history, serology results, and imaging evidence of spinal involvement or favorable treatment response^8–11^. Typically, clinical characteristics encompass undulant fever, weakness,fatigue, lombago, and discernible neurological impairment. However, due to the widespread usage of antibiotics in recent years, the proportion of patients exhibiting the classical clinical presentation of brucellosis has diminished. Hence, the significance of epidemiological exposure history and diagnostic evaluations has grown considerably. Epidemiological exposure history primarily centers around the consumption of unpasteurized dairy products derived from infected animals or occupational exposures^9,12^. Diagnostic laboratory assessments and imaging modalities primarily encompass the Rose Bengal plate test (RBPT), serum agglutination test (SAT), lumbar radiography, computed tomography (CT), and magnetic resonance imaging (MRI). The RBPT serves as an initial screening tool for brucellosis patients, while SAT titers exceeding 1:100 are employed for definitive diagnosis. Nonetheless, the possibility of misdiagnosing brucella spondylitis as tuberculosis (TB) spondylitis exists, underlining the criticality of employing imaging techniques for differential diagnosis when confronting these two types of spinal infections^13–16^. Erdem et al. suggested that in the absence of microbiological results, a constellation of constitutional symptoms such as fever, back pain, weight loss, pulmonary involvement, elevated inflammatory markers, and radiological findings can aid in early-stage differentiation between TB spondylitis and Brucella spondylitis^14^.

Antibiotics are widely perceived to be the cornerstone of brucellosis treatment. Some studies have conducted comparisons to evaluate the efficacy of various antibiotic regimens in managing spinal brucellosis^17–23^. Our study employed a therapeutic approach founded on the administration of standard antimicrobial agents (rifampicin and doxycycline), augmented by appropriate surgical intervention. According to Katonis et al., surgical treatment should be considered as a final recourse in cases of spinal brucellosis, given the typically favorable response to chemotherapy^24^. In cases where patients with LBS present with severe intervertebral disc destruction, cartilage endplate defect, spinal instability, progressive neurological functional deterioration, or kyphosis deformity, a combined approach of surgical intervention and antimicrobial chemotherapy would be more suitable. However, the optimal surgical procedure for LBS remains a subject of debate. The traditional method involves a one-stage anterior debridement, bone grafting, and posterior instrumentation, but this approach is associated with drawbacks such as prolonged surgical duration, technical complexity, and the need for intraoperative positional adjustments. In recent years, there has been an increasing adoption of a one-stage posterior pedicle fixation, debridement, and interbody fusion technique as researchers delve further into the study of Brucella spondylitis^25–27^. Nevertheless, there is limited literature available that directly compares the clinical effectiveness of the two surgical methods for managing LBS. Consequently, in this study, we conducted a comparative analysis of the follow-up data of patients with LBS who underwent a one-stage posterior procedure versus those who underwent an anterior and posterior approach. The aim was to assess and compare the efficacy of these two surgical techniques.

## Materials and methods

### Patient population

Inclusion criteria were as follows: (1) patients with confirmed *Brucella* infection, defined as patients with positive *Brucella* culture or SAT test, a minimum antibody titer > 1:100; (2) persistence of low back pain despite 6 weeks of pharmaceutical treatment; (3) presence of diverse levels of bone destruction, paravertebral abscess or psoas abscess, degenerative changes in the intervertebral discs, and necrosis as observed through imaging; (4) functional spinal units involving ≤ 2 segments; and (5) a follow-up period exceeding 12 months.

Exclusion criteria were as follows: (1) patients with a prior history of pulmonary tuberculosis; (2) metastatic tumor of the spine; (3) contraindication to surgery, such as unsatisfactory overall health condition for anesthesia; (4) lack of complete follow-up data; (5) history of previous lumbar surgery; (6) patients diagnosed as LBS but with a lesser severity than that mentioned in the inclusion criteria.

According to the above inclusion and exclusion criteria, from January 2015 to June 2020, a total of 62 patients with LBS were retrospectively analyzed. Group A included 33 cases (15 men and 18 women with a mean age of 44.6 ± 13.5 years) who were treated with a one-stage posterior pedicle fixation, debridement, and interbody fusion. Group B comprised 29 patients (14 men and 15 women with a mean age of 46.3 ± 14.9 years) who were treated with a posterior pedicle fixation combined with anterior retroperitoneal debridement and interbody fusion. The selection of the surgical procedure was based on X-rays, CT scans, and MRI of the affected lumbar area, which were examined to determine the location, severity, and extent of the lesion.

### Preoperative preparation

All patients received anti-brucellosis chemotherapy, including oral administration of doxycycline (100 mg, every 12 h) and rifampicin (600 mg/day, daily) for at least 7 days before surgery. The surgical procedure was conducted once the patient achieved afebrile status and the anemia and hypoproteinemia were resolved.

### Surgical procedures

#### One-stage posterior pedicle fixation, debridement, and interbody fusion

In group A, general endotracheal anesthesia and the prone position were adopted. The spinous process of the affected vertebrae was identified and marked using C-arm lateral fluoroscopy, and a longitudinal incision was then made along the midline of the diseased vertebrae. The paravertebral muscles were dissected subperiosteally to expose the vertebral laminae and facet joints of the affected segments. Posterior pedicle screws were inserted to provide temporary stability. To create a larger operative space for lesion clearance, the spinous process, upper and lower laminae, and ligamentum were excised using a rongeur or piezosurgery technique. An interbody fusion cage filled with osseous granules from healthy lamina bone was created. Different curettes were used to remove the sequestrum and necrotic disc tissue meticulously, and osteotomes were used to remove sequestrum around the lesion. Subsequently, an interbody fusion cage was implanted for interbody fusion. Titanium rods were inserted to provide stabilization and correct any kyphosis. A drainage tube was appropriately placed to ensure optimal wound drainage, and meticulous closure of the incision was carried out. The duration of the surgical procedure and the amount of intraoperative blood loss were meticulously recorded. A biopsy specimen was promptly sent for both bacterial culture and pathological diagnosis purposes. *(2)* Anterior debridement, bone grafting and posterior instrumentation.

All patients in group B underwent posterior pedicle fixation utilizing identical techniques employed in group A. Subsequently, the patient's position was altered to the supine or lateral position. The retroperitoneal approach was adopted for the anterior debridement and interbody fusion. Likewise, the surgical level was accurately determined under fluoroscopic guidance. An precise incision was made at the entry point on theabdominal skin and continued through the underlying subcutaneous tissue. Following meticulous dissection of the subcutaneous tissue, the anterior rectus sheath was incised, and the rectus muscle was delicately mobilized medially.The posterior rectus sheet was opened and the peritoneum was exposed and carefully retracted using a hand. With meticulous dissection, the surrounding vasculature was carefully separated upon reaching the anterior edge of the vertebral body. Complete removal of paravertebral, psoas major, and anterior vertebral margin abscesses or inflammatory granuloma tissue after full visualization of the lesioned segment, followed by complete removal of the damaged intervertebral disc, dead bone, and sclerotic bone within the vertebral body was performed. An iliac allograft block of appropriate dimensions was prepared and implanted. A drainage tube was appropriately positioned within the affected area, and the incision was closed layer by layer. The biopsy specimens were processed in the same manner.

### Postoperative care

Vital signs, including temperature and lower extremity movements and sensory perception, were closely monitored. Prophylactic antibiotics were administered for 48–72 h, and the same anti-*Brucella* protocol was continued for at least 6 weeks, terminating at least 2 weeks after a negative RBPT. Sensitive antibiotics were changed over time according to the drug sensitivity results, and liver and kidney functions were checked regularly once a month during the medication period. The drainage tube was removed after the drainage volume was less than 20 mL/day. After drainage removal, the patients were encouraged to gradually increase physical activity under the protection of braces, and bed rest was recommended for at least three months after the operation. Any perioperative complications were duly documented.

### Evaluation indexes and follow-up actions

All postoperative evaluations were performed at the outpatient clinic of our hospital. The follow-up interval was every three months within the first year after surgery and every six months within the second year after surgery. The operation duration, intraoperative blood loss, average length of hospital stay, and perioperative complications were recorded. Follow-up items included blood parameters (erythrocyte sedimentation rate [ESR] and C-reactive protein [CRP]), SAT test to assess the activity of *Brucella*, and imaging manifestations to evaluate whether the lesion was healed or recurred. The visual analog scale (VAS) score, lordosis angle, time of bone graft fusion, Oswestry disability index (ODI) score, and the Japanese Orthopaedic Association (JOA) score were also recorded to assess clinical efficacy.

### Statistical analysis

The SPSS 26.0 (IBM, USA) statistical software was used for data analysis. A repeated measures ANOVA was used to analyze the date of pre-, postoperative and final follow-up. The paired-samples t-test was used to analyze data between two groups, any discrepancy in normal distribution was analyzed using the rank sum test and *P* < 0.05 was considered to be significantly different.

### Ethics approval and consent to participate

This study protocol was approved by the Ethics Committee of Xiangya Hospital (No:202206151). We also followed the Declaration of Helsinki and its later amendments. Written informed consent was obtained from all patients.

## Results

All cases were followed up at least 2 years with an average of 25.4 ± 1.5 months (range 24–30 months). All surgical procedures were executed successfully, resulting in complete resolution of lumbar brucella spondylodiscitis (LBS) in each patient. Throughout the hospitalization period and subsequent follow-up, no instances of clinical or radiological relapse were observed. In group A, one case experienced superficial wound infection, while in group B, one case each experienced intraoperative peritoneal rupture, postoperative ileus, iliac vein injury, and superficial wound infection. There were no significant disparities in age or in the preoperative, three-month postoperative (TMP), and final follow-up (FFU) measurements of the visual analog scale (VAS) score, erythrocyte sedimentation rate (ESR), C-reactive protein (CRP), and time required for the serological agglutination test (SAT) to achieve negative status between the two groups (*P* > 0.05). (Table 1).

**Table 1:** Basic clinical data and evaluation indexes comparison of each group.

Basic clinical data	Group A	Group B	*P* value
Age (years)	44.6 ± 13.5	46.3 ± 14.9	0.666
Sex (M/F)	19/14	17/12	–
Follow-up time (months)	25.4 ± 1.4	25.5 ± 1.7	0.881
VAS			
Preoperative	7.81 ± 1.0	7.79 ± 0.9	0.885
Three months postoperative	1.24 ± 0.6*	1.17 ± 0.7*	0.206
Final follow-up	0.97 ± 0.8*	0.97 ± 0.9*	0.869
ESR (mm/h)			
Preoperative	64.4 ± 28.4	60.0 ± 34.1	0.456
Three months postoperative	28.5 ± 20.4*	28.9 ± 18.0*	0.393
Final follow-up	12.2 ± 4.1*	11.9 ± 4.6*	0.975
CRP (mg/L)			
Preoperative	21.9 ± 19.6	22.2 ± 19.4	0.951
Three months postoperative	12.1 ± 10.7*	13.5 ± 13.4*	0.842
Final follow-up	7.7 ± 3.2*	7.6 ± 3.2*	0.945
Lordosis angle (°)			
Preoperative	16.1 ± 2.0	17.2 ± 1.6	0.251
Three months postoperative	28.0 ± 1.8*	27.8 ± 2.0*	0.544
Final follow-up	28.6 ± 2.1*	28.5 ± 1.8*	0.350
ODI score			
Preoperative	76.6 ± 2.3	76.1 ± 2.5	0.448
Three months postoperative	23.3 ± 2.2*	23.7 ± 2.3*	0.650
Final follow-up	4.1 ± 2.0*	4.0 ± 2.4*	0.905
Operation time (mins)	78.6 ± 11.3	103.6 ± 16.4	0.000
Intraoperative blood loss (mL)	81.8 ± 19.4	117.2 ± 23.2	0.000
Fusion time (months)	7.6 ± 0.8	7.3 ± 0.8	0.165
Time of SAT transfer to negative (months)	5.9 ± 1.2	6.1 ± 1.1	0.161
Average length of stay (days)	15.1 ± 1.9	18.3 ± 1.6	0.000

*Analyzed by repeated measures ANOVA, compared with preoperatively, *P* < 0.05.

The average operative duration in group A was 78.6 ± 11.3 min, and the mean intraoperative blood loss amounted to 81.8 ± 19.4 mL. Both of these measurements were significantly lower compared to group B, which recorded respective values of 103.6 ± 16.4 min and 117.2 ± 23.2 mL (*P* < 0.05). The average length of hospital stay for group B was 18.3 ± 1.6 days, which exhibited a significant increase when compared to group A's duration of 15.1 ± 1.9 days. (*P* < 0.05) (Table 1).

The ODI scores of groups A and B improved significantly at the TMP (23.3 ± 2.2 and 23.7 ± 2.3, respectively, *P* > 0.05) and continued to improve at the FFU (4.1 ± 2.0 and 4.0 ± 2.4, respectively, *P* > 0.05), which were significantly lower than the preoperative scores (76.6 ± 2.3 and 76.1 ± 2.5, respectively, *P* > 0.05) (Table 1).

The JOA scores of both groups A and B improved significantly at the TMP (21.1 ± 2.1 and 21.5 ± 2.3, respectively, *P* > 0.05) and continued to improve, with JOA scores at the FFU of 25.9 ± 0.9 and 26.2 ± 0.9, respectively (*P* > 0.05), which were significantly higher than the preoperative score of 7.8 ± 1.4 and 8.1 ± 1.5, respectively, (*P* > 0.05). In addition, we used the JOA score rating system to calculate neurological improvement with the following formula: neurological improvement rate (IR) = (post-treatment score $-$ pre-treatment score) / (29 $-$ pre-treatment score) × 100%. Remarkable improvements in neurological function were observed for each patient. The IR of the JOA scores of groups A and B at the TMP were 62.6% ± 10.4% and 64.2% ± 11.0%, respectively, which increased to 85.2% ± 4.9% and 86.6% ± 4.6%, respectively, at the FFU (Table 2).

**Table 2:** JOA score (29) for neurological status evaluation.

JOA score	Group A	Group B	*P* value
Preoperative	7.8 ± 1.4	8.1 ± 1.5	0.134
Three months postoperative	21.1 ± 2.1*	21.5 ± 2.3*	0.656
Improvement rate of TMP (%)	62.6 ± 10.4	64.2 ± 11.0	0.689
Final follow-up	25.9 ± 0.9*	26.2 ± 0.9*	0.110
Improvement rate of FFU (%)	85.2 ± 4.9	86.6 ± 4.6	0.125

*Analyzed by repeated measures ANOVA, compared with preoperatively, *P* < 0.05.

*TMP* three months postoperative, *FFU* final follow-up.

**Figure 1:**
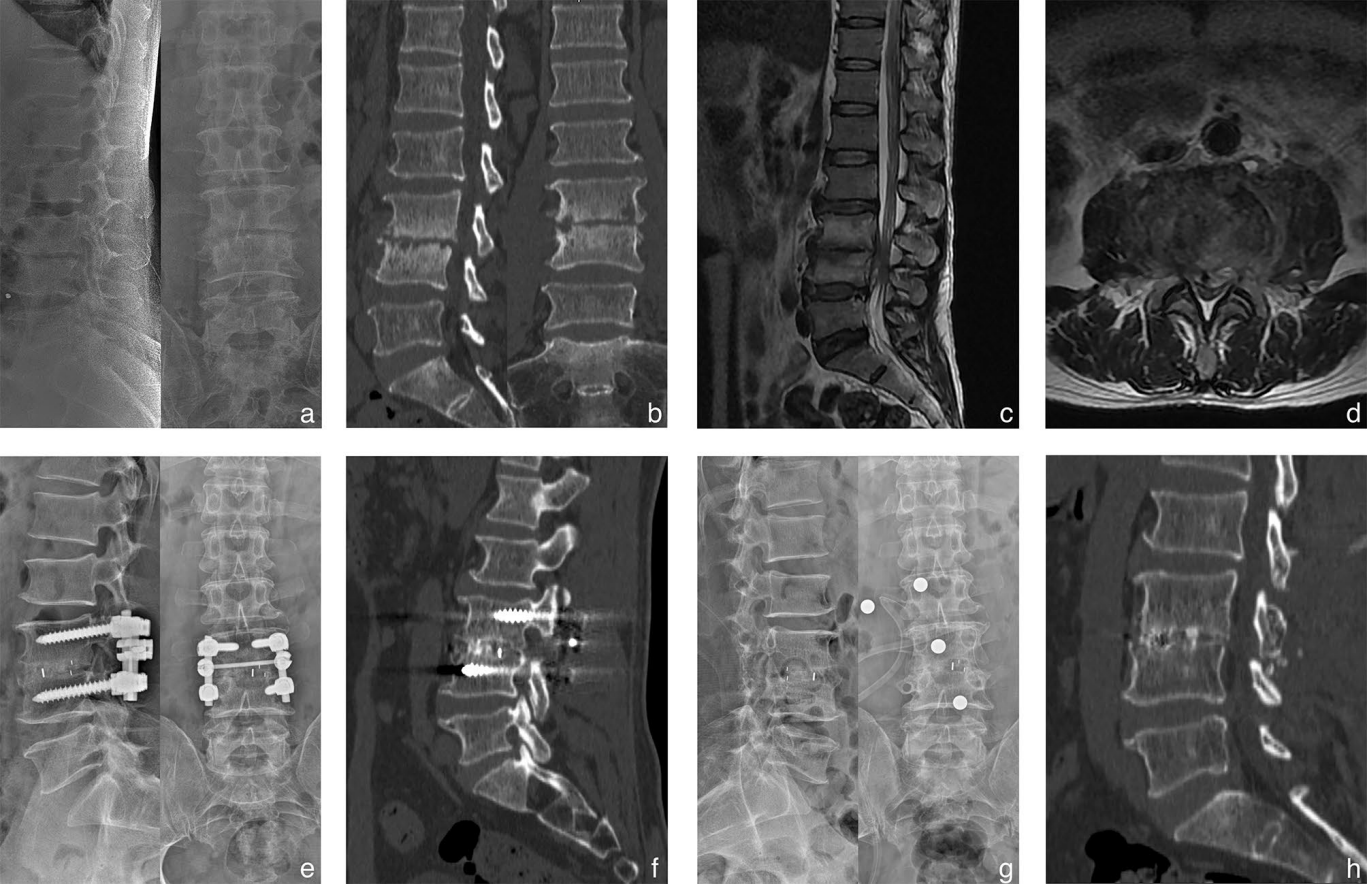
A 53-year-old male shepherd diagnosed with L3-4 brucella spondylitis underwent one-stage posterior interbody fusion and debridement procedure. Preoperative X-ray revealed intervertebral height loss and significant narrowing of the affected disc space. Preoperative CT and MRI scans exhibited erosions of the superior and inferior endplates, along with evidence of nerve compression. (**a**–**d**) Postoperative X-ray and CT images demonstrated satisfactory positioning of the internal fixation and cage. (**e**–**f**) The final follow-up conducted at 26 months indicated successful bone fusion following the removal of instrumentation, as evidenced by radiographic and CT examinations (**g**–**h**).

**Figure 2:**
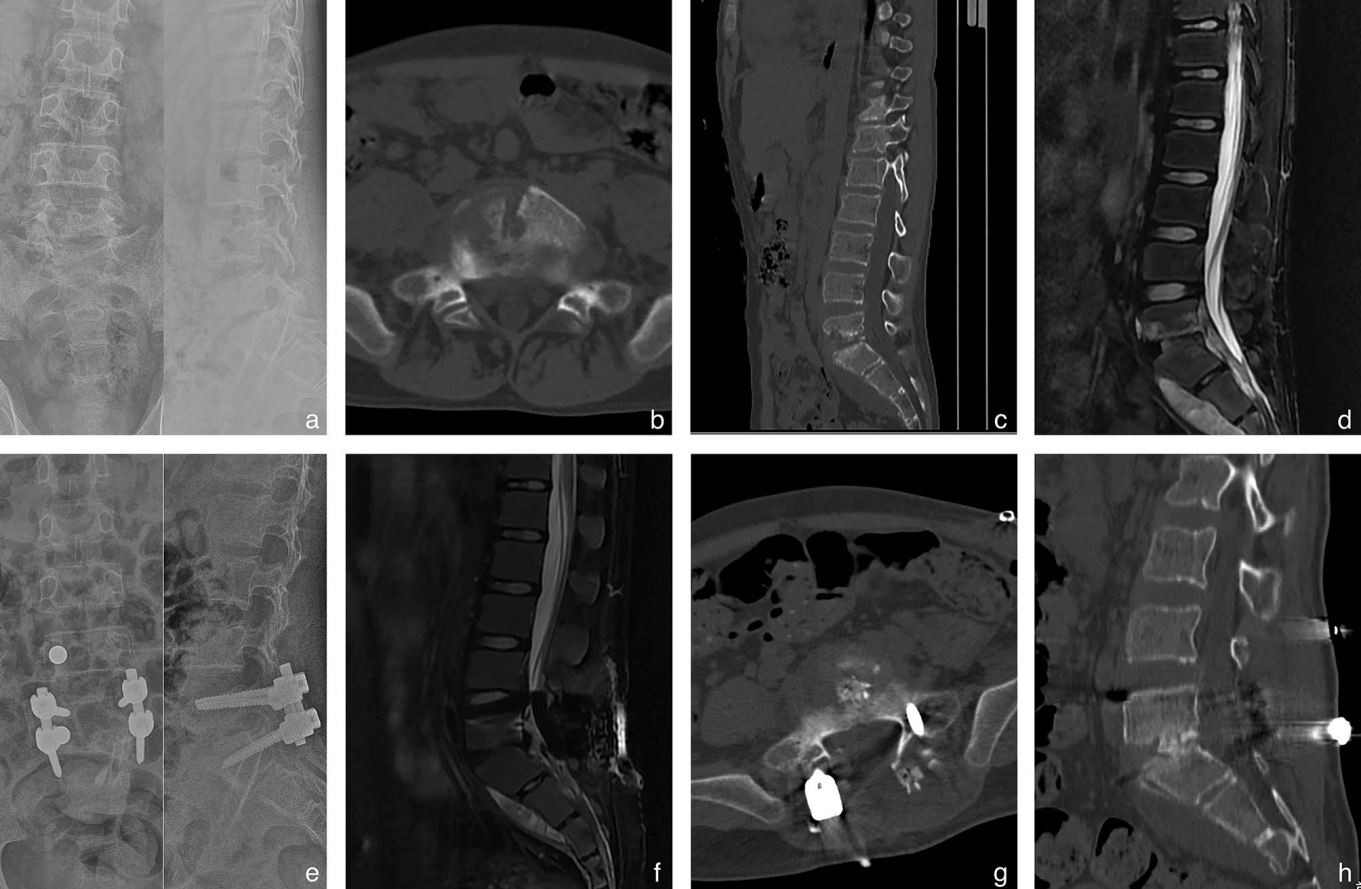
A 22-year-old female patient diagnosed with L5-S1 brucella spondylitis underwent a comprehensive treatment approach consisting of posterior pedicle fixation combined with anterior retroperitoneal debridement and interbody fusion. Preoperative anteroposterior and lateral X-ray, CT, and MRI scans revealed intervertebral stenosis, as well as significant disc and vertebral body destruction. (**a**–**d**) The postoperative imaging materials exhibited successful resolution of the infection and confirmed solid bony fusion during the final follow-up (**e**–**h**).

The average lordosis angles of groups A and B at the TMP increased to 28.0° ± 1.8° and 27.8° ± 2.0°, respectively from 16.1° ± 2.0° and 17.2° ± 1.6°, respectively. At the FFU, the correction was not lost, and the lordosis angle of the two groups was 28.6° ± 2.1° and 28.5° ± 1.8°, respectively, which significantly improved in comparison with the preoperative measurements. The mean time of bone graft fusion was 7.6 ± 0.8 and 7.3 ± 0.8 months in group A and B respectively (*P* > 0.05) (Figs. 1 and 2).

## Discussion

### Necessity and indications of surgery for Brucella spondylitis

The lumbar region is the most commonly involved site of Brucella spondylitis, which is a significant contributor to debilitating and incapacitating complications^5,12,20,28^. The systemic management of lumbar brucellosis, including the duration of antibiotic therapy, selection of appropriate antibiotic combinations, and choice of surgical approaches, lacks standardization, and and the indications and contraindications for various surgical approaches remain a subject of controversy. Several experts advocate for surgical intervention in patients who experience persistent systemic infection symptoms, progressive neurological deterioration, and spinal instability despite chemotherapy treatment. This positive surgical intervention aims to alleviate pain, eradicate the infectious focus, ameliorate neurological dysfunction, correct spinal deformity, and restore spinal stability^11,24^. Thus, in accordance with relevant literature^5,17,20,21,26,29,30^, we propose the following indications for surgical intervention in the treatment of brucellosis spondylitis: (1) definite diagnosis of brucellosis spondylitis and completion of two standard courses of chemotherapy, resulting in improvement of systemic toxicity symptoms but no alleviation or even exacerbation of severe low back pain. (2) severe intervertebral disc destruction and cartilage endplate defect with spinal instability or severe destruction and collapse of vertebral structures leading to spinal deformity; (3) vertebral canal abscess or inflammatory granuloma compressing the spinal cord, cauda equina, or nerve roots, causing significant neurological symptoms. (4) presence of a large non-resorbable paravertebral or psoas abscess; and (5) Coexistence of mixed infection with other bacteria.

### Selection of surgical approach

One-stage anterior debridement, fusion, and fixation are considered reasonable approaches for LBS. According to Yin et al., a retrospective cohort study demonstrated that one-stage anterior debridement, utilization of autogenous grafts, and instrumentation can be effective and feasible treatment modalities for LBS without involvement of the posterior column^29^. However, the anterior approach also possesses inherent drawbacks. For instance, unstable fixation of the vertebral body may lead to compromised fusion of bone grafts, as the strength of vertebral body fixation is inferior to that of pedicle screws. Consequently, some experts acknowledge the posterior approach, as it enables three-dimensional correction via posterior pedicle screw fixation traversing all three spinal columns. Chen et al. reported favorable efficacy and safety outcomes with one-stage debridement, utilization of autogenous bone grafts, and posterior instrumentation^26^. Hence, this technique presents a potential alternative for addressing lumbar brucellosis spondylitis (LBS) in cases involving cauda equina syndrome, radiculopathy, spinal instability, and severe back pain triggered by extradural nonabsorbable abscess or progressive collapse. Nonetheless, both of these studies were limited by the absence of comparative control studies with larger cohorts. Na et al. stated that both the anterior and posterior approaches effectively achieve remission of LBS, yet the posterior approach offers superior correction of kyphotic deformity, reduced surgical invasiveness, and decreased incidence of complications^30^.

Anterior debridement and bone graft fusion combined with posterior internal fixation are the common surgical methods for lumbar spondylitis^31,32^. However, this combination surgical method for LBS treatment has rarely been reported or compared with the other two common approaches.

### Comparison of two kinds of surgical approach

#### Characteristics of anterior and posterior combination surgical procedure

In Group B, 29 patients underwent posterior pedicle fixation combined with anterior retroperitoneal debridement and interbody fusion.

The advantages of this approach are as follows: (1) anterior debridement allows for complete scraping of vertebral bone destruction, paravertebral abscesses or psoas abscess, and intervertebral disc lesions under direct vision and elimination of dead space to realize bone defect repair and spinal cord decompression; (2) anterior intervertebral bone grafting can reconstruct anterior and middle column heights, avoid kyphosis, and support the unstable spine; and (3) posterior internal fixation overcomes the difficulty of grasping the quality of the vertebral body in anterior fixation, which could lead to loosening of the internal fixation and loss of correction and allow the implant to be placed relatively distant from the lesion, reducing the chance of postoperative infection and facilitating fusion of the implants. Thus, this approach has inherent advantages for patients with large paravertebral or psoas major abscesses and severe kyphosis.

However, the combined anterior and posterior surgical procedure necessitates a lengthier operation time, exhibits higher invasiveness, and leads to increased blood loss. Moreover, given that brucellosis is a chronic debilitating condition, most patients are already moderately or severely malnourished prior to surgery, resulting in a heightened anesthetic risk and a potential for severe anterior complications during the postoperative stage. Consequently, many patients are unable to tolerate this particular procedure. Autologous iliac crest bone grafting was performed on all patients in our study. It is worth noting that donor site morbidity and prolonged pain following iliac crest bone graft harvesting have been well-documented in orthopedic surgery. Notably, the mean length of hospital stay, average duration of the operation, intraoperative blood loss, postoperative visual analog scale (VAS) score, and the mean Oswestry Disability Index (ODI) score at 3 months of follow-up were significantly higher compared to the posterior approach. These findings indicate that the posterior approach not only inflicts less trauma compared to the combined anterior and posterior approaches but also necessitates lower surgical tolerance requirements and results in faster patient recovery.

#### Characteristics of the posterior surgical procedure

In Group A, 33 patients underwent posterior pedicle fixation, debridement, and interbody fusion. This approach offers inherent benefits such as the correction of kyphotic deformity and stabilization of the spine. Additionally, the following advantages are associated with this technique: (1) it circumvents the elevated anesthetic risk associated with anterior procedures, which may lead to significant postoperative complications; (2) the combination of posterior internal fixation, debridement, and interbody fusion can be accomplished through a single incision, aligning with the principles of "minimally invasive" surgery; and (3) there is no requirement for positional changes during the operation, resulting in shorter surgery duration and minimal trauma.

Nevertheless, the posterior approach has limitations such as restricted visibility and a narrow surgical field, making it challenging to achieve complete clearance of bony lesions, large paravertebral abscesses, or psoas major abscesses within the anterior vertebral body through direct visualization alone. To address this, C-arm X-ray guidance and discoscopic assistance are necessary. Failure to thoroughly eradicate these conditions could lead to postoperative recurrence. Brucella spondylitis is characterized by infectious inflammation of the vertebral column and intervertebral discs, with abscess formation being less common compared to spinal tuberculosis. As a result, a posterior-only procedure may be more suitable for the majority of patients with Brucella spondylitis. However, patients with extensive paravertebral abscesses or significant anterior spinal column destruction would not be suitable candidates for a purely posterior approach.

Within our study, all patients met clear indications for surgical intervention and were managed using either a one-stage posterior approach or a combined one-stage anteroposterior approach. Multiple evaluation parameters were employed to assess the procedure's effectiveness, and all patients exhibited satisfactory clinical outcomes. The functional follow-up (FFU) revealed significant improvements in erythrocyte sedimentation rate (ESR), C-reactive protein (CRP) levels, visual analog scale (VAS) scores, and Oswestry Disability Index (ODI) scores in both groups when compared to the values at the time of preoperative treatment (TMP). Radiographic findings indicated that all cases achieved successful graft fusion without apparent signs of recurrence. Consequently, both techniques demonstrated efficacy in the treatment of lumbar spondylitis (LBS). Nevertheless, it is important to note that our study was retrospective in nature and encompassed a limited number of cases. Therefore, these conclusions should be further validated through a multicenter prospective randomized controlled study.

## Conclusions

Provided that the indications for surgery are strictly adhered to, both surgical interventions can be used to treat LBS with satisfactory results. In cases where patients present with extensive paravertebral and/or psoas major abscesses or substantial structural bone damage in the anterior vertebral column, a combined anterior–posterior approach is more appropriate. Conversely, patients predominantly affected by brucellosis, those with compromised general health and underlying conditions, and individuals displaying posterior spinal column pathology, particularly those with notable disc destruction or spinal nerve compression within the spinal canal, are better suited for the less invasive posterior approach. Additionally, the posterior approach offers distinct advantages such as shorter surgical duration, reduced blood loss, briefer hospital stays, and fewer perioperative complications. Consequently, the one-stage posterior pedicle fixation, debridement, and interbody fusion is a superior treatment option.

## Data Availability

The datasets and materials generated or analyzed during the current study are available from the corresponding author on reasonable request.

## Abbreviations

CRP: C-reactive protein
CT: Computed tomography
ESR: Erythrocyte sedimentation rate
FFU: Final follow-up
JOA: Japanese Orthopaedic Association
LBS: Lumbar Brucella spondylitis
MRI: Magnetic resonance imaging
ODI: Oswestry disability index
RBPT: Rose Bengal Plate Test
SAT: Serum agglutination test
TMP: Three-month postoperative
TB: Tuberculosis
VAS: Visual analog scale
